# Synergistic Effect and Molecular Mechanism of Homoharringtonine and Bortezomib on SKM-1 Cell Apoptosis

**DOI:** 10.1371/journal.pone.0142422

**Published:** 2015-11-06

**Authors:** Jing Zhang, Bobin Chen, Ting Wu, Qian Wang, Lin Zhuang, Chen Zhu, Ni Fan, Wenjiao Qing, Yan Ma, Xiaoping Xu

**Affiliations:** Department of Hematology, Huashan Hospital, Shanghai Medical College, Fudan University, Shanghai, China; Emory University, UNITED STATES

## Abstract

**Background:**

Myelodysplastic syndromes (MDS) are clonal marrow stem-cell disorders with a high risk of progression to acute myeloid leukemia (AML). Treatment options are limited and targeted therapies are not available for MDS. In the present study, we investigated the cytotoxicity and the molecular mechanism of Homoharringtonine (HHT) and Bortezomib towards high-risk MDS cell line SKM-1 *in vitro* and the role of miR-3151 was first evaluated in SKM-1 cells.

**Methods:**

SKM-1 cells were treated with different concentrations of HHT or Bortezomib, and cell viability was analyzed with CCK-8 assay. The influence on cell proliferation, cell cycle distribution and the percentage of apoptosis cells were analyzed by flow cytometry. Calcusyn software was used to calculate combination index (CI) values. Western blot was used to analysis phosphorylation of Akt and nuclear NF-κB protein expression in SKM-1 cells. Mature miR-3151 level and p53 protein level were detected after HHT or Bortezomib treatment. The cell proliferation and p53 protein level were reassessed in SKM-1 cells infected with lentivirus to overexpress miR-3151.

**Results:**

Simultaneous exposure to HHT and Bortezomib (10.4:1) resulted in a significant reduction of cell proliferation in SKM-1 cells (P < 0.05). Cell cycle arrest at G0/G1 and G2/M phase was observed (P < 0.05). HHT and Bortezomib synergistically induced cell apoptosis by regulating members of caspase 9, caspase 3 and Bcl-2 family (P < 0.01). The mechanisms of the synergy involved Akt and NF-κB signaling pathway inhibition, downregulation of mature miR-3151 and increment of downstream p53 protein level. Overexpression of miR-3151 promoted cell proliferation and inhibited p53 protein expression in SKM-1 (P < 0.01).

**Conclusions:**

HHT and Bortezomib synergistically inhibit SKM-1 cell proliferation and induce apoptosis *in vitro*. Inhibition of Akt and NF-κB pathway signaling contribute to molecular mechanism of HHT and Bortezomib. miR-3151 abundance is implicated in SKM-1 cell viability, cell proliferation and p53 protein expression.

## Introduction

Myelodysplastic syndromes (MDS) are clonal marrow stem-cell disorders, characterized by ineffective haemopoiesis leading to blood cytopenias. One third of patients have a risk of acute myeloid leukemia (AML). Median survival in patients classified as high or intermediate 2 on the International Prognostic Scoring System (IPSS) are only about 12 months [1]. Patients with MDS who progress to AML have shorter durations of complete remission (CR) than the ones with de-novo AML [1, 2]. Treatment of MDS remains challenging. Allogeneic hematopoietic stem cell transplantation remains the only curative treatment of high-risk MDS. However, comorbidities and impaired functional status lead to poor treatment outcome. Therefore, innovative approaches for patients with high-risk MDS are still necessary.

Homoharringtonine (HHT), an antitumor plant alkaloid, has been widely used in China to treat patients with AML since 1970s. A number of clinical studies have confirmed a significant effect of HHT on AML [3–5]. Patients with AML who received induction therapy consisting of HHT and cytarabine (HA) have similar CR rates compared to combinations of anthracyclines and cytarabine [6]. HHT based triple drug combinations, especially the HAA regimen are highly effective in the treatment of *de novo* AML [7]. The HAG priming regimen (G-CSF priming combined with low-dose HA chemotherapy) is effective and safe as an induction therapy for patients, including elderly patients, with high-risk MDS and MDS/AML, with CR rates of 46.7%~57.6% in China [8, 9]. Wu et al [10] reported the median overall survival (OS) of elderly patients with high-risk MDS or MDS/AML receiving the HAG regimen as induction chemotherapy was 15 months. These results reflect that more effective alternative regimens are necessary to improve the outcome of patients with high-risk MDS and MDS/AML, although scattered and retrospective clinical trials in China have confirmed the important role of HHT in high-risk MDS and MDS/AML.

Since NF-κB activation is responsible for the progressive suppression of apoptosis affecting differentiation of MDS cells and contribute to malignant transformation [11], proteasome inhibitors may be effective in the treatment of high-risk MDS. Bortezomib is a proteasome and NF-κB inhibitor that can induce cell apoptosis *in vitro* and has demonstrated benefit when used as a single agent in high-risk MDS patients [12, 13]. The mechanisms involved decreased activation of the PI3K/Akt survival signaling pathway and were related to inhibition of the NF-κB activity and downregulation of the Bcl-2/Bax ratios [13].

The expression of miR-3151 is activated by the SP1/NF-κB complex and can be inhibited by Bortezomib. Eisfeld et al [14] reported that high expression of miR-3151 is an independent prognosticator for poor outcome in cytogenetically normal AML (CN-AML). Increase of miR-3151 reduced the cell apoptosis and chemosensitivity of AML cell lines and increased leukemogenesis in mice. miR-3151 bound to the 3′untranslated region of *TP53* to disrupt the *TP53*-mediated apoptosis pathway in AML cells [15]. However, the role of miR-3151 and NF-κB in high-risk MDS cell line SKM-1 has not been reported.

Riccioni et al [16] reports that patients with AML-M4 and AML-M5 are more sensitive to Bortezomib than other FAB classification. HHT is also highly effective in the treatment of AML-M5 [17, 18]. In addition, HHT enhances cytotoxicity of Bortezomib against multiple myeloma cells concomitantly with inhibition of phosphorylated Akt [19]. In the present study, we investigated the cytotoxicity and the molecular mechanism of HHT and Bortezomib towards high-risk MDS cell line SKM-1 *in vitro* and the role of miR-3151 was first evaluated in SKM-1 cells.

## Materials and Methods

### Cell line and reagents

The high-risk MDS cell line SKM-1 was purchased from the Health Science Research Resources Bank in Japan, and cultured in RPMI-1640 medium (Gibico) supplemented with 10% fetal bovine serum (Gibico) in a 37°C, 5% CO_2_ incubator. Normal human peripheral blood mononuclear cells (PBMC) were isolated from a healthy volunteer donor, from whom written formed consent for experimental use was obtained. The protocols received approval from the ethics committees of the Institutional Review Board of Huashan Hospital, Fudan university (Permit Number: 2011–037). Dilute blood 1:1 with calcium-magnesium-free PBS and layer 4 ml onto 4 ml Ficoll-Paque PREMIUM (GE Healthcare). Centrifuge at 400×g for 20 minutes at 20°C. Draw off the upper layer without disturbing the interface. Collect the interface with a cannula and diluted to RPMI-1640. HHT was obtained from Minsheng Pharma (Hangzhou, China). Bortezomib was obtained from Xian-Janssen Pharmaceutical Ltd. (Xian, Shanxi, China).

### Cell viability assays

Cell viability was measured by CCK-8 assay (Beyotime, Shanghai, China) following manufacturer’s instructions. 10 μL CCK-8 solution was added to each well and the plates were incubated for an additional 4 h at 37°C. The absorbance at 450 nm was measured. The percentage of viable cells was calculated using the formula: ratio (%) = [OD (Treatment)–OD (Blank)] / [OD (Control)–OD (Blank)] × 100. Control group was cell culture medium containing same concentration of drugs. Each sample was assayed with six replicates per assay, each experiment was carried out in three repeats. The effects of sequential exposure to HHT and Bortezomib were assessed by exposing cells to graded concentrations of drugs at fixed ratios. The schedules tested were as follows: (a) HHT for 24 h and then Bortezomib for 24 h; (b) Bortezomib for 24 h and then HHT for 24 h; and (c) HHT and Bortezomib for 48 h. Serial dilutions of drug concentrations were done as follows: HHT (1/4, 1/2, 1, 2, 4×IC50) and Bortezomib (1/4, 1/2, 1, 2, 4×IC50). The median effect lines were analyzed by the method of Chou and Talalay [20].

### Proliferation assay and cell cycle analysis

Proliferation assay and cell cycle analysis was determined using the BrdU Flow Kits (BD Pharmingen) following manufacturer’s instructions. Add 10 μL of BrdU solution (1 mM) directly to each mL of tissue culture medium and incubate the treated cells for 45 min. Fix and permeabilize the cells with BD Cytofix/Cytoperm Buffer and BD Cytoperm Permeabilization Buffer Plus. Then treat cells with DNase to expose incorporated BrdU. Stain BrdU and intracellular antigens with fluorescent antibodies. Stain total DNA for cell cycle analysis. Cells were analyzed on a FACScan flow cytometer (BD Biosciences) and data were interpreted using the Flowjo software (Treestar).

### Cell apoptosis analysis

Cell apoptosis was determined using the Annexin V Apoptosis Detection Kit APC (eBioscience) following manufacturer’s instructions. Add 5 μL of fluorochrome-conjugated Annexin V to 100 μL of the cell suspension. Incubate 15 min at room temperature. Wash cells in 1× Binding Buffer and resuspend in 200 μL of 1× Binding Buffer. Add 5 μL of Propidium Iodide Staining Solution Analyze by flow cytometry within 4 h. Cells were analyzed on a FACScan flow cytometer (BD Biosciences) and data were interpreted using the Flowjo software (Treestar).

### Analysis of nuclear morphology

Cells were fixed by incubating in stationary liquid for 15 min at room temperature. After being washed twice with PBS, cells were permeabilized with 0.2% Triton X-100 for 30 min, washed with PBS, stained with 10 μg / mL Hoechst 33342 (Sigma) for 30 min, and washed with PBS again. Nuclear morphology was observed immediately using a fluorescence microscope ((Nikon, Tokyo, Japan).

### Western blot analysis

Cells following treatment were washed twice in PBS and lysed with RIPA (Beyotime, Shanghai, China) in the presence of the protease inhibitor PMSF (Beyotime, Shanghai, China). Protein concentration was determined using the BCA protein assay kit (Beyotime, Shanghai, China). Protein samples were separated by SDS-PAGE and transferred to PVDF membranes (Immobilon-P membrane; Millipore, USA). After incubation in 5% fat-free milk at room temperature for 2 h to block non-specific binding, membranes were incubated with primary antibodies at 1:1000 dilution in TBS-Tween (TBST) (0.05% Tween-20 in TBS) at 4°C overnight. Membranes were then washed three times with TBST and incubated with secondary antibody at 1:5000 dilution for 1 h at room temperature. Membranes were again washed three times with TBST and visualized by enhanced chemiluminescence using Supersignal West Pico Trial Kit (Thermo Scientific, Massachusetts, USA).

### Quantitative real-time PCR (qRT-PCR)

Total RNA was extracted using Trizol Reagent (Invitrogen) following manufacturer’s instructions. Reverse transcription and qRT-PCR was performed using the All-in-One™ miRNA qRT-PCR Detection Kit (GeneCopoeia Inc.) on a 7500 Real-Time PCR System (ABI, Grand Island, NY, USA). All reactions were performed in three repeats. miR-3151 and U6 primers used for qRT-PCR were purchased from GeneCopoeia Inc.

### Lentivirus infection

Lentivirus vectors were constructed by Bioeasy (Shanghai, China). Each vector contains a target gene hsa-miR-3151 and a green fluorescent protein (GFP) marker. SKM-1 was transfected with recombinant lentivirus-miR-3151 expression vector (miR-3151 precusor) in a complete medium using polybrene. Cells transfected with lentiviral pLVX-eGFP-IRES-Puro vector without clones of pre- miR-3151 were used as controls. After 48 h of transfection, GFP expression was examined using flow cytometry.

### Statistical analysis

The half-maximal inhibitory concentrations (IC50) were calculated using GraphPad Prism (GraphPad Software, USA). The combination index (CI) was calculated using the Chou–Talalay method (Calcusyn software, Biosoft, San Diego, CA, USA) to ascertain if the effects of drug combinations were synergistic (CI < 1), additive (CI = 1), or antagonistic (CI > 1) [20]. Results are presented as means ± standard deviation (SD). The dose–response analyses were performed by one-way ANOVA with Dunnett’s-t test. All statistical analyses were performed using the software Stata 9.0 for windows. P < 0.05 was considered significant.

## Results

### HHT and Bortezomib synergistically inhibit growth of SKM-1 cells *in vitro*

**Fig 1:**
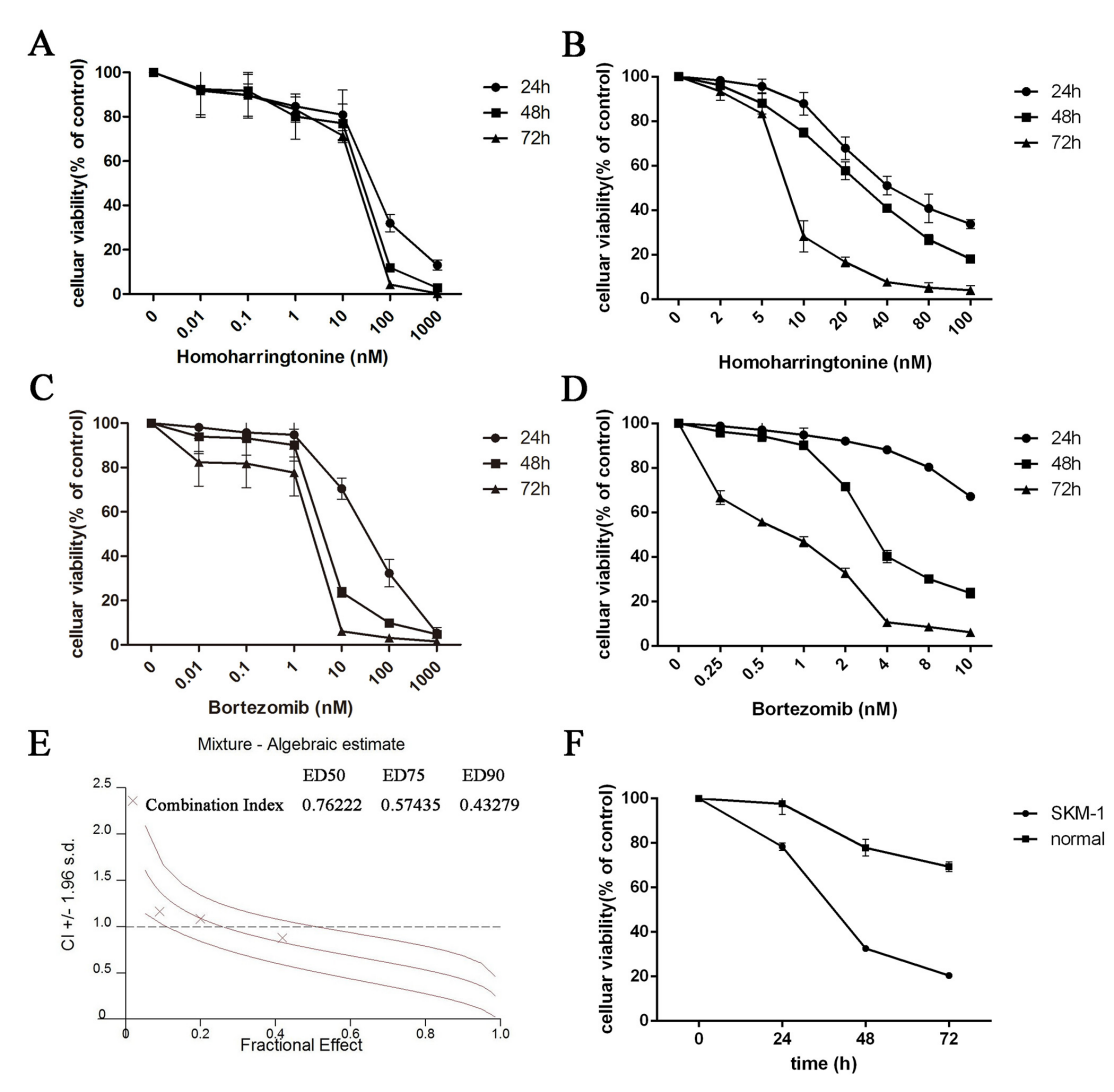
HHT and Bortezomib inhibited the cell viability of SKM-1 cells. (A) Time- and dose–response curves of HHT from 0.01 nM to 1000 nM. (B) Time- and dose–response curves of HHT from 2 nM to 100 nM. (C) Time- and dose–response curves of Bortezomib from 0.01 nM to 1000 nM. (D) Time- and dose–response curves of Bortezomib from 0.25 nM to 10 nM. (E) CI values for HHT and Bortezomib combination treatments at the molar ratio of 10.4:1. (F) Celluar viability of SKM-1 or normal PBMC cells simultaneously treated with 46.20 nM HHT and 4.46 nM Bortezomib.

SKM-1 cells were plated into 96-well plates and treated with increasing concentrations of HHT (0.01, 0.1, 1, 10, 100 and 1000 nM) or Bortezomib (0.01, 0.1, 1, 10, 100 and 1000 nM) for 24 h, 48 h and 72 h. The CCK-8 assay showed a time and dose-dependent cell viability inhibition by HHT and Bortezomib (Fig 1A, 1B, 1C and 1D, P < 0.01). The IC50 values for HHT in SKM-1 cells at 24 h, 48 h and 72 h were 43.65 ± 4.37 nM, 23.10 ± 3.38 nM and 17.62 ± 6.89 nM, respectively. The IC50 values for Bortezomib in SKM-1 cells at 24 h, 48 h and 72 h were 34.21 ± 5.30 nM, 4.46 ± 4.26 nM and 2.116 ± 8.05 nM, respectively. The dose–effect curves for HHT + Bortezomib were determined by Calcusyn analyses. We found that simultaneous exposure to HHT and Bortezomib for 48 h resulted in a strong synergistic inhibition of cell viability in SKM-1 cells. The best synergistic effect was observed when the molar ratio was 10.4: 1 (HHT: Bortezomib). The CI value at the median effective dose (ED50) was 0.76222 (Fig 1E). The inhibition of cell growth in SKM-1 was more significant than normal PBMC cells simultaneously treated with 46.20 nM HHT and 4.46 nM Bortezomib (Fig 1F).

**Fig 2:**
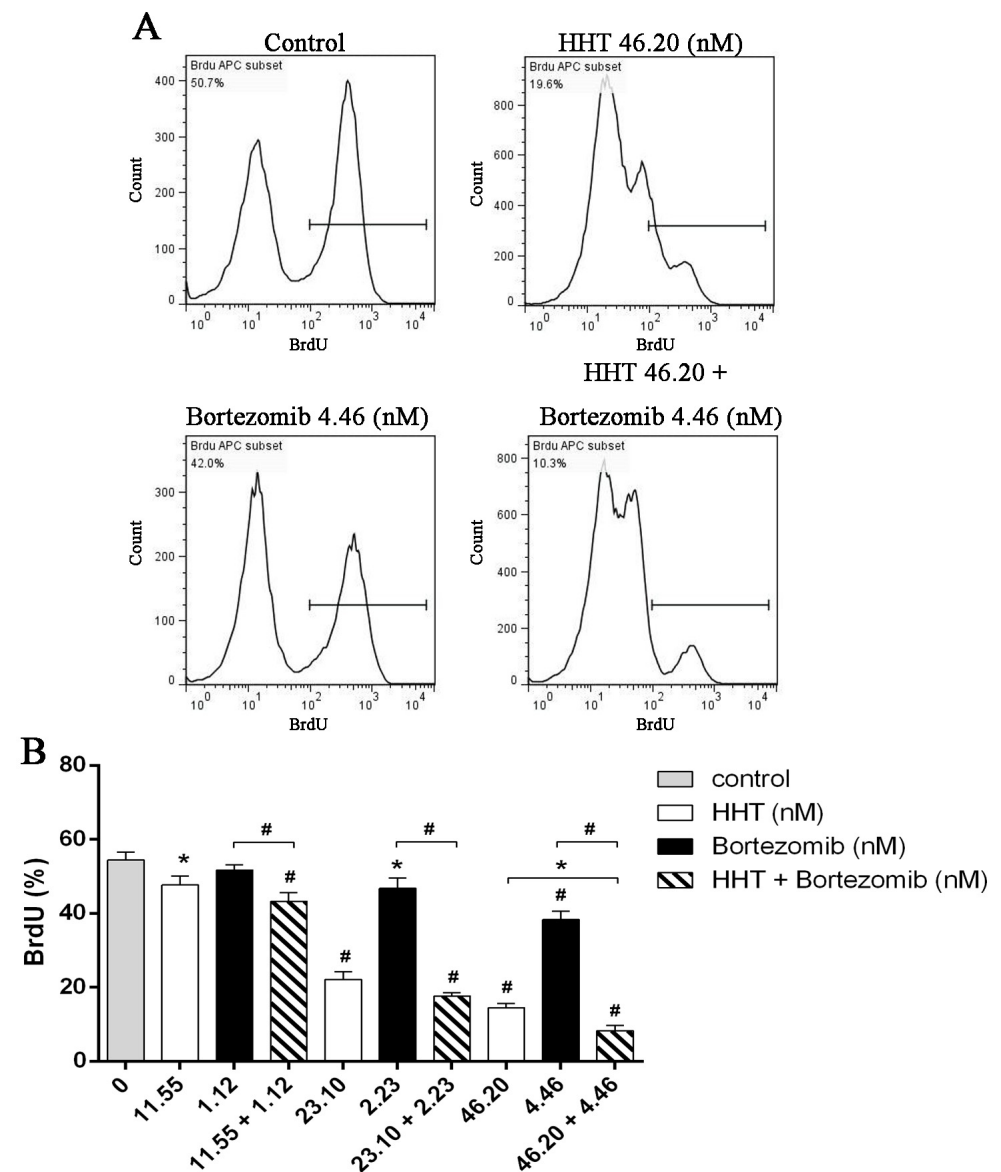
Simultaneous exposure to HHT and Bortezomib resulted in a significant reduction of cell proliferation. (A) SKM-1 cells treated with 46.20 nM HHT, 4.46 nM Bortezomib or 46.20 nM HHT + 4.46 nM Bortezomib simultaneously for 72 h. (B) SKM-1 cells treated with HHT (11.55, 23.10, 46.20 nM) or Bortezomib (1.12, 2.23, 4.46 nM) for 72 h. Concurrently, simultaneous treatment experiments were conducted at molar ratio of 10.4: 1. Bar graph of BrdU positive rates.*P < 0.05, #P < 0.01 vs untreated control.

**Fig 3:**
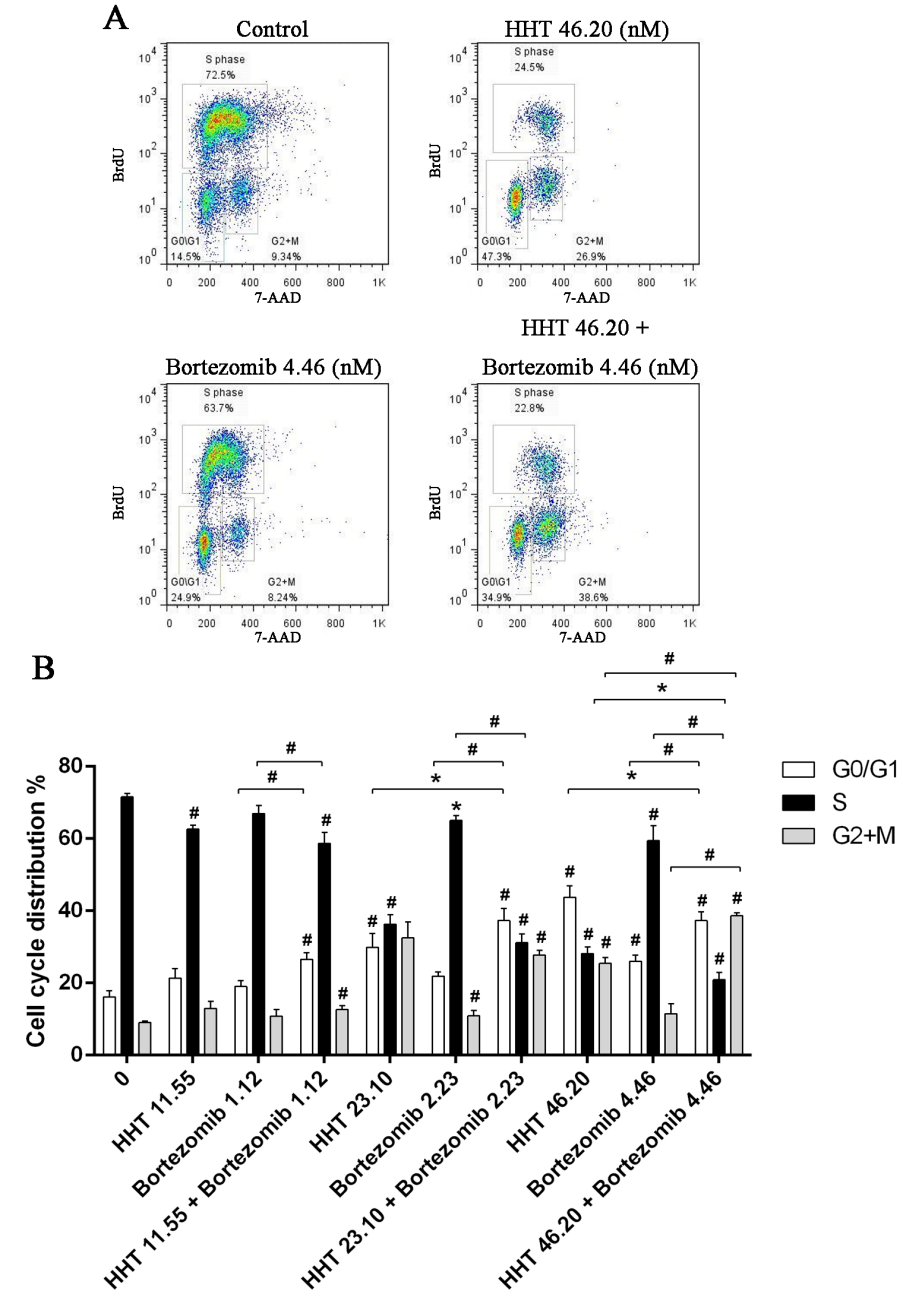
Simultaneous exposure to HHT and Bortezomib resulted in cell cycle arrest. (A) SKM-1 cells treated with 46.20 nM HHT, 4.46 nM Bortezomib or 46.20 nM HHT + 4.46 nM Bortezomib simultaneously for 72 h. (B) SKM-1 cells treated with HHT (11.55, 23.10, 46.20 nM) or Bortezomib (1.12, 2.23, 4.46 nM) for 72 h. Concurrently, simultaneous treatment experiments were conducted at molar ratio of 10.4: 1. Bar graph of cell cycle distribution. *P < 0.05, #P < 0.01 vs untreated control.

We further examined the effects of HHT (11.55, 23.10, 46.20 nM) and Bortezomib (1.12, 2.23, 4.46 nM) on SKM-1 cell proliferation and cell cycle distribution using BrdU/7AAD double staining flow cytometry. Similar to results from the cell viability assay, simultaneous exposure to HHT and Bortezomib (10.4: 1) for 72 h resulted in a significant reduction of cell proliferation in SKM-1 cells (Fig 2, P<0.05). Cell cycle arrest at G0/G1 and G2/M phase was observed in SKM-1 cells incubated with different concentrations of HHT or Bortezomib for 72 h, which was accompanied by a decreased proportion at S phase (Fig 3, P<0.05). It was more obvious when HHT: Bortezomib was 10.4:1. Collectively, these results indicated that HHT and Bortezomib synergistically inhibited the growth of SKM-1 cells *in vitro*.

### HHT and Bortezomib synergistically induces cell apoptosis by regulating members of caspase 3 and Bcl-2 family

**Fig 4:**
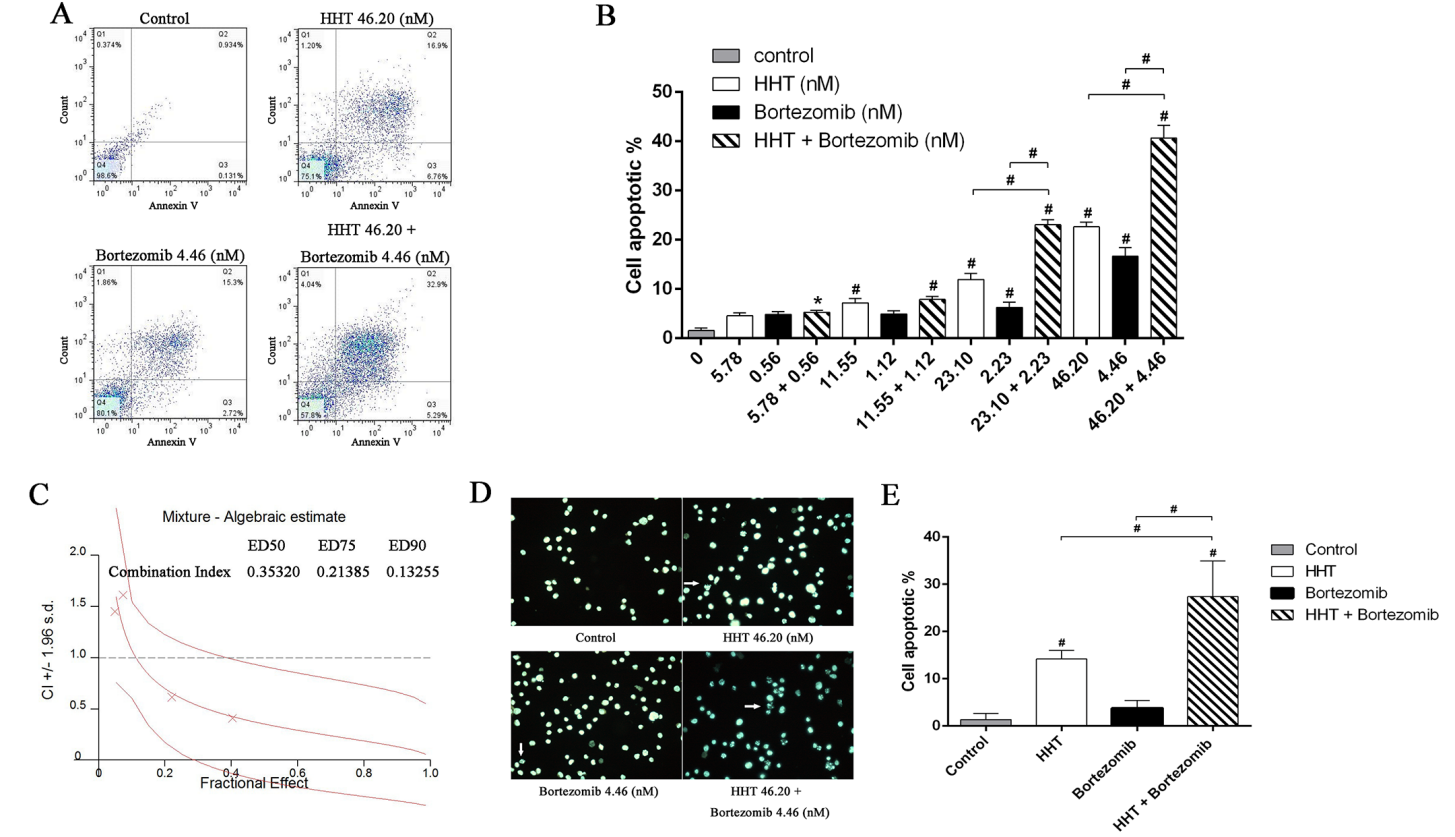
HHT and Bortezomib synergistically induced apoptosis of SKM-1 cells. (A) Annexin V/PI double-staining flow cytometry of SKM-1 cells treated with 46.20 nM HHT, 4.46 nM Bortezomib and simultaneous treatment experiments at molar ratio of 10.4: 1. (B) The proportion of apoptotic cells in a histogram. (C) CI values for HHT and Bortezomib combination treatments at the molar ratio of 10.4:1. (D) Cell apoptosis assessed by florescence microscopy (magnification 400×). The arrows indicate the cells undergoing apoptosis. (E) The percent of apoptotic cells determined by morphology-based cell sorting. *P < 0.05, #P < 0.01 vs untreated control.

The results of Annexin V/PI assay indicated that apoptosis rates increased with increasing drug concentration treated with HHT (5.78, 11.55, 23.10, 46.20 nM) or Bortezomib (0.56, 1.12, 2.23, 4.46 nM), mainly in the late apoptosis and a dose-dependent manner (Fig 4B, P < 0.01). Compared with single agents, a combination of HHT and Bortezomib (10.4:1) resulted in a significant increase in apoptosis. The CI values at ED50, ED75, ED90 were 0.35320, 0.21385 and 0.13255, respectively (Fig 4C). After treatment with 46.20 nM HHT alone, 4.46 nM Bortezomib alone, or combination of HHT and Bortezomib, SKM-1 cells were stained with Hoechst 33342 to observe nuclear morphology. As shown in Fig 4D, cell shrinkage, chromatin condensation and fragmentation was observed by florescence microscopy. A higher number of SKM-1 cells with apoptotic morphology was observed by fluorescence microscopy in the combined group than in the single-agent groups (Fig 4E, P < 0.01).

**Fig 5:**
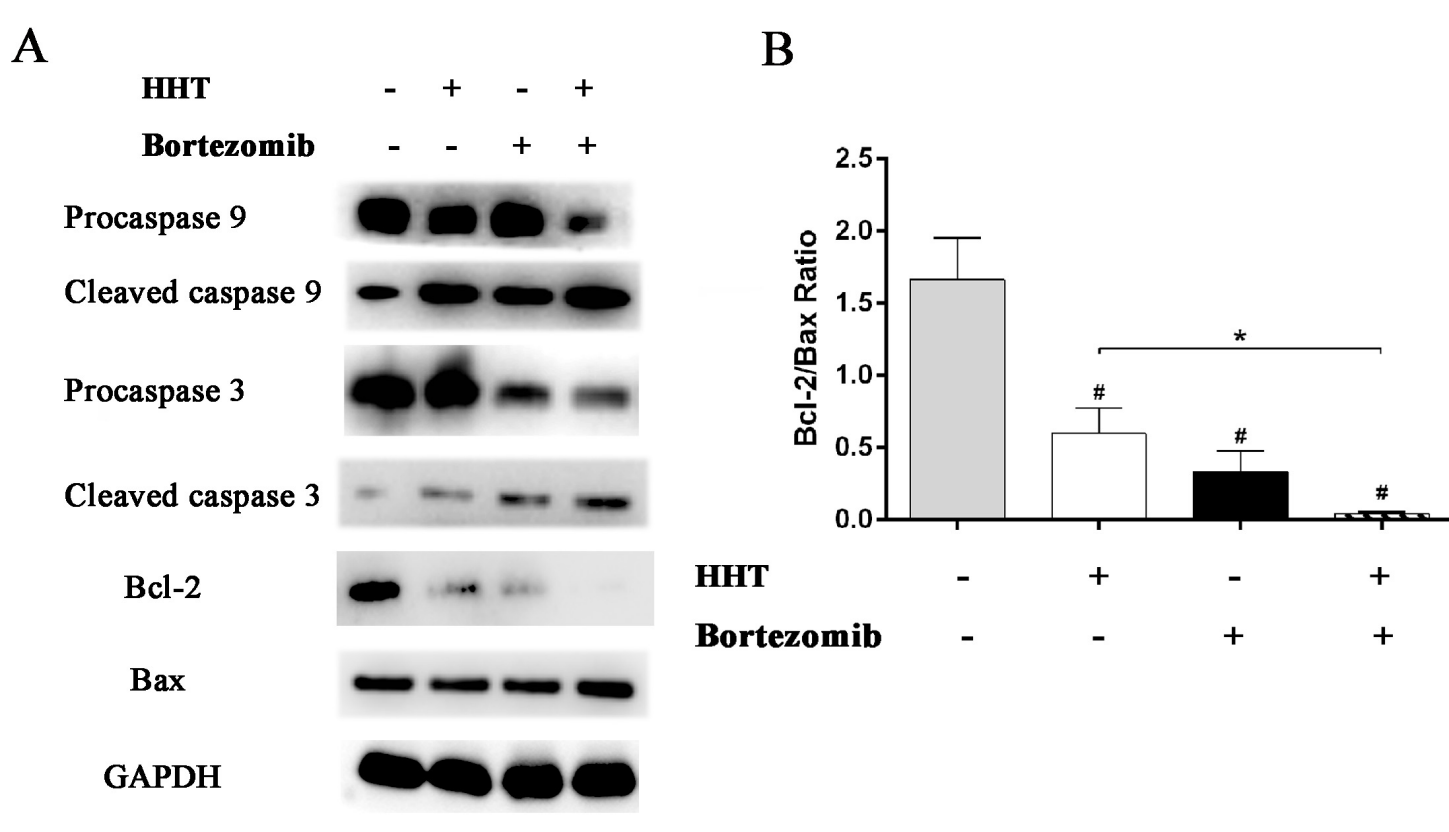
Effects of HHT and Bortezomib on caspases and Bcl-2 family proteins. HHT and Bortezomib simultaneously actived caspase 9 and caspase 3, induced the up-regulation of Bax protein and the down-regulation of Bcl-2 protein after 72 h exposure. *P < 0.05, #P < 0.01 vs untreated control.

Western blotting was used to evaluate the expression of caspase 9, caspase 3, Bcl-2 and Bax in SKM-1 cells after treatment with HHT, Bortezomib or combination of HHT and Bortezomib for 72 h. Caspase 9 and caspase 3 are important members of the caspase family. Cleaved caspase 9 further processes caspase 3 to initiate a caspase cascade leading to apoptosis. The results showed that 11.55 nM HHT or 1.12 nM Bortezomib significantly increased cleavage activation of caspase 9 and caspase 3, inhibited anti-apoptotic protein Bcl-2 expression and upregulated pro-apoptotic protein Bax. The effect of drugs was more obvious when HHT: Bortezomib was 10.4:1 (Fig 5).

### HHT and Bortezomib cooperate to inhibit Akt and NF-κB pathway signaling in SKM-1 cells

**Fig 6:**
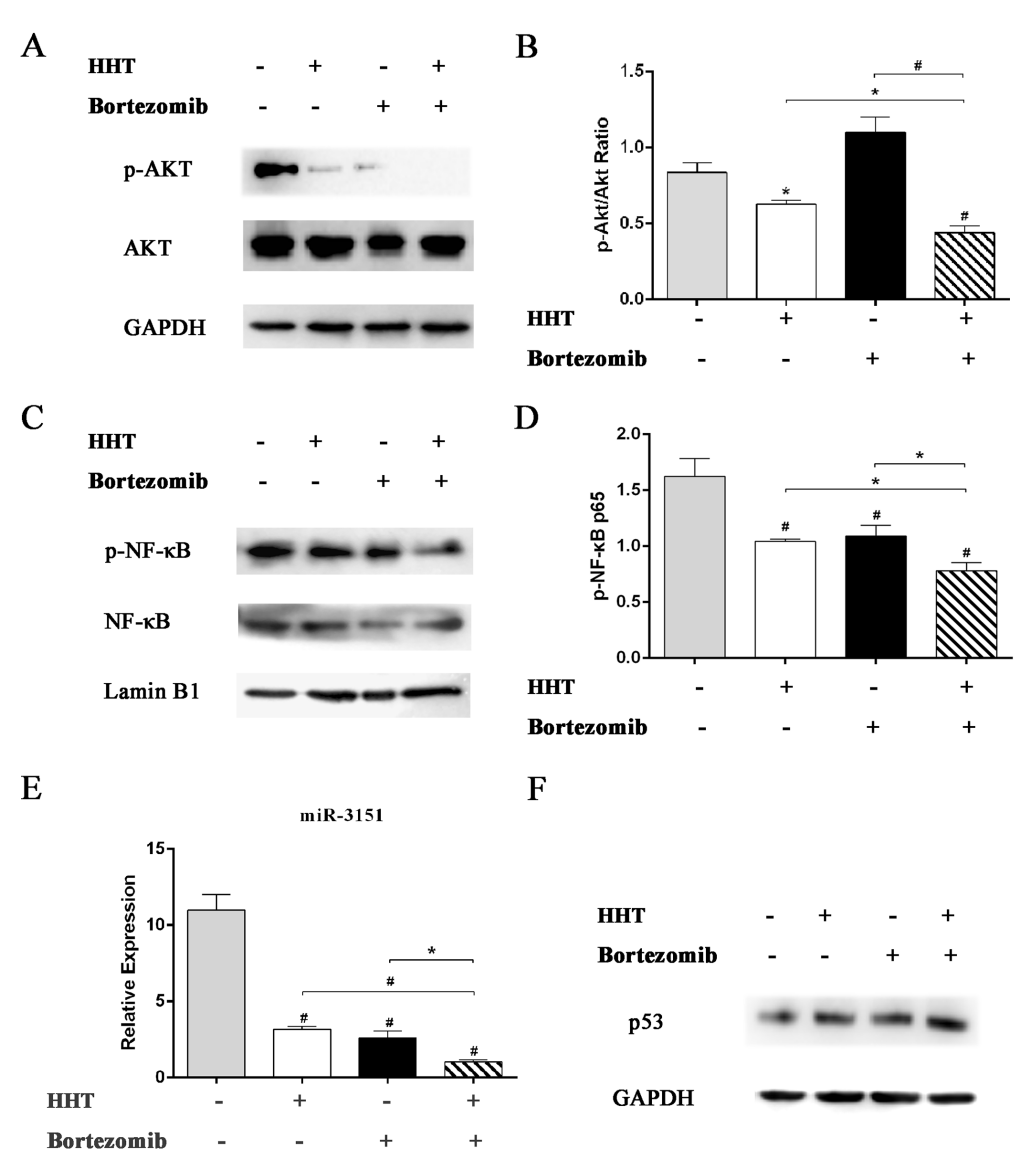
Effects of HHT or Bortezomib on Akt and NF-κB signaling pathway. Western blot analyses of p-Akt, Akt (A and B), p-NF-κB, NF-κB (C and D) and p53 (F) protein expression in SKM-1 cells exposed to 11.55 nM HHT or 1.12 nM Bortezomib for 72 h. (E) Real-time PCR detection of miR-3151 expression level in SKM-1 cells exposed to 11.55 nM HHT or 1.12 nM Bortezomib for 6 h. *P < 0.05, #P < 0.01 vs untreated control.

To further investigate the synergetic mechanism of HHT and Bortezomib, we analyzed the effects of HHT and Bortezomib on the proliferative and survival signals Akt and NF-κB in SKM-1 cells. Abnormal activation of Akt and NF-κB signaling pathways have been reported in MDS. Inhibition of these two signaling pathways can inhibit cell proliferation and increase apoptosis in MDS. As shown in Fig 6, HHT and Bortezomib reduced p-Akt and p-NF-κB expression in SKM-1 cells. Combination of HHT and Bortezomib at 10.4:1was more effective compared with single agents. Our results suggested that the synergistic inhibition of HHT and Bortezomib in SKM-1 cell proliferation was related to inhibition of Akt and NF-κB signaling pathway. Furthermore, 11.55 nM HHT or 1.12 nM Bortezomib significantly inhibited the expression of mature miR-3151(Fig 6E, P < 0.01). Compared with single agents, a combination of HHT and Bortezomib resulted in a significant decrease of miR-3151. HHT and Bortezomib also increased downstream p53 protein level in SKM-1 cells (Fig 6F).

### Overexpression of miR-3151 promotes cell proliferation and inhibition of p53 protein expression in SKM-1

**Fig 7:**
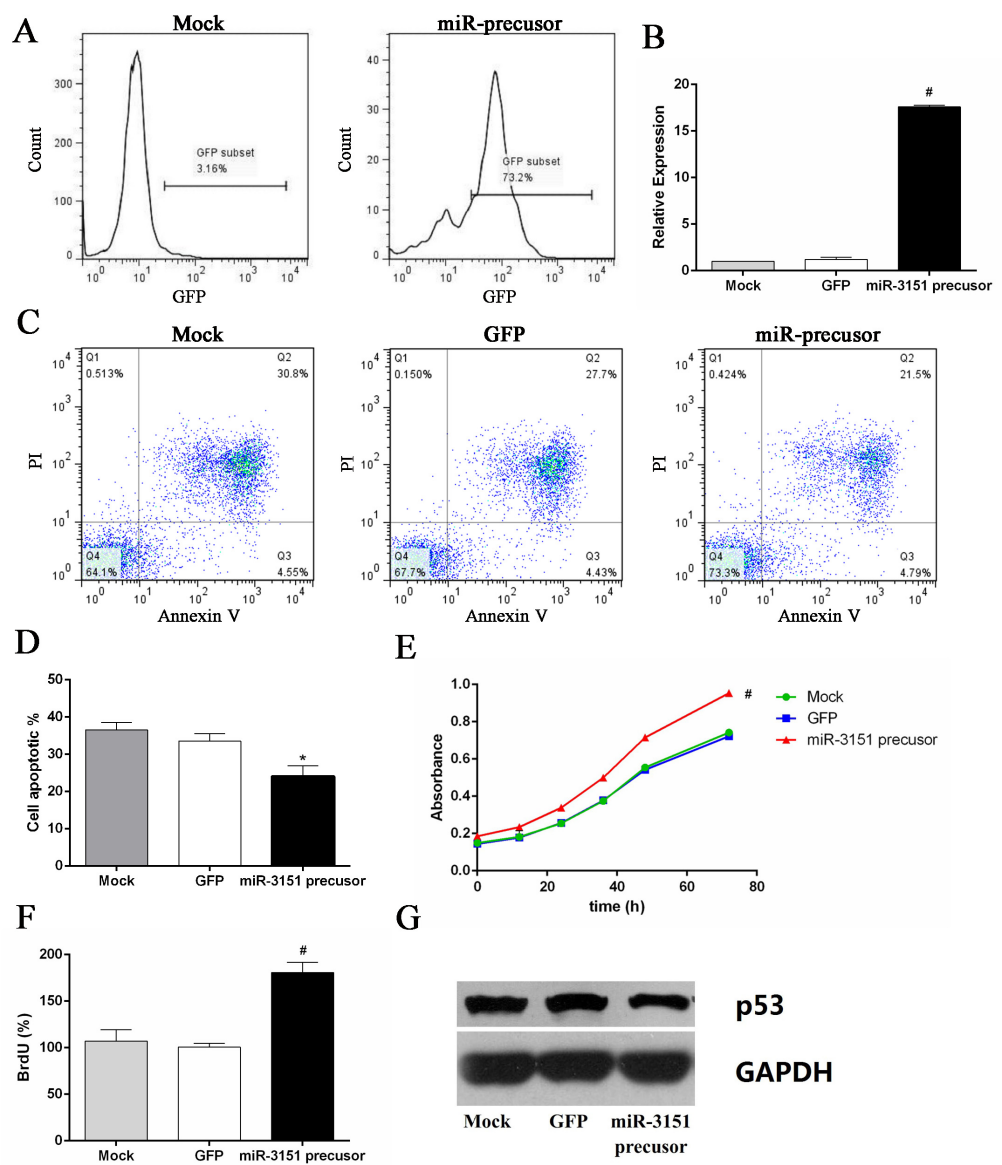
Overexpression of miR-3151 promoted cell proliferation and inhibition of p53 protein expression. (A) The positive rate of GFP in miR-3151 precusor group cells. (B) miR-3151 relative expression level after transfection. (C) Annexin V/PI double-staining flow cytometry of SKM-1 cells overexpressing miR-3151 treated with 46.20 nM HHT and 4.46 nM Bortezomib. (D) Overexpressing miR-3151 of SKM-1 cells partly reversed the effect of HHT and Bortezomib. (E) The cell viability of SKM-1 cells overexpressing miR-3151. (F) The cell proliferation of SKM-1 cells overexpressing miR-3151. (G) p53 protein level in SKM-1 cells overexpressing miR-3151. *P < 0.05, #P < 0.01 vs mock group.

Over 70% transfection efficiency was observed in SKM-1 cells after 48 h transfection (Fig 7A). The cell viability and proliferation of SKM-1 cells overexpressing miR-3151 were significantly higher than the control group, accompanied by decreased p53 protein level. Overexpressing miR-3151 of SKM-1 cells partly reversed the effect of HHT and Bortezomib (Fig 7, P < 0.01).

## Discussion

In this study, we showed that HHT or Bortezomib alone resulted in an inhibition of cell growth and promotion of apoptosis in SKM-1 cells in a dose-dependent manner. To extend our analysis of the synergistic interactions of HHT and Bortezomib, different ratios of HHT and Bortezomib were used to treat SKM-1 cells. Cell viability assays showed a synergistic anti-proliferative effect, as indicated by the CIs at molar ratios of 10.4: 1 (HHT: Bortezomib). Cells were arrested in G0/G1 and G2/M phase. Similarly, combination of HHT with Bortezomib strongly and synergistically induced apoptosis in SKM-1 cells. A high Bcl-2/Bax ratio is associated with poor prognosis and decreased rates of CR and OS [21, 22]. HHT and Bortezomib treatment resulted in a strong downregulation of Bcl-2 and up-regulation of Bax in SKM-1 cells, decreasing the Bcl-2/Bax ratio. Downregulation of the Bcl-2/Bax ratio and the activation of caspase cascades contributes to cell apoptosis [23]. The activation of caspase 9 and caspase 3 were observed in SKM-1 cells treated with HHT and Bortezomib. Caspase 9 and caspase 3 protein are members of the cysteine-aspartic acid protease (caspase) family, which play a central role in the execution-phase of cell apoptosis [24]. Initiator caspase (caspase 9) cleave and activate inactive pro-forms of effector caspase (caspase 3). caspase 3 in turn cleave other protein substrates within SKM-1 cells to trigger the apoptotic process.

Constitutive activation of NF-κB might significantly contribute to the pathogenesis of high-risk MDS [12]. The anti-tumor effect of Bortezomib involves inhibition of NF-κB activity, altered degradation of cell cycle proteins, altered balance of pro and anti-apoptotic proteins and DNA repair [25]. To elucidate the molecular mechanisms involved in the synergistic effects of HHT and Bortezomib, the expression of phosphorylated Akt and NF-κB was evaluated in SKM-1 cells treated with HHT and Bortezomib. The results indicated that the synergistic mechanisms of HHT and Bortezomib were related to decreased activation of Akt and involved inhibition of the NF-κB activity. Akt activation is one of the factors contributing to the decreased apoptosis rate in high-risk MDS [26]. Downstream of Akt, NF-κB has been associated with several tumorigenesis mechanisms, including promoting cancer cell proliferation, preventing cell apoptosis, increasing angiogenesis, cancer-related inflammation and metastatic potential [27, 28].

In order to further investigate the mechanism of synergistic cytotoxicity induced by HHT/Bortezomib combination in SKM-1 cells, downstream miR-3151 abundance and p53 expression were assessed. Numerous studies have been undertaken to successfully provide evidence for an involvement of miRNAs in the regulation of proliferation, differentiation, apoptosis and tumorigenesis [29]. Research on the function of miR-3151 is still limited. Eisfeld et al [14] first reported that the expression of miR-3151 was activated by the SP1/NF-κB complex in AML cell line. Overexpressing miR-3151 in leukemia cells disrupts the *TP53*-mediated apoptosis pathway. p53 regulates several key processes to suppress cancer, including cell-cycle arrest, DNA repair, apoptosis and senescence [30, 31]. Plenty of experimental results indicate that miRNAs are important to the p53 network [32–34]. p53 regulates the expression and processing of miRNAs, but is also under the control of certain miRNAs, such as miR-3151. miR-3151 abundance can be reduced by pharmacologic inhibition of SP1/NF-kB binding in AML cell line. In our research, HHT and Bortezomib reduced miR-3151 abundance and increased p53 expression. Furthermore, we found that overexpression of miR-3151 promoted cell proliferation and inhibited p53 expression in SKM-1. Our preliminary study of miR-3151 in high-risk MDS cell line suggested that miR-3151 might play an important role in progression to AML. It is still unclear whether miR-3151 could be used as a prognostic indicator in patients with high-risk MDS. Therefore, further investigation on miR-3151 in high-risk MDS is necessary.

In summary, we demonstrate that HHT and Bortezomib synergistically inhibits SKM-1 cell proliferation and induces apoptosis *in vitro*. The mechanism of synergy involves Akt and NF-κB signaling pathway inhibition, mature miR-3151 downregulation and downstream p53 protein level increase. Overexpression of miR-3151 enhances SKM-1 cell viability, accelerates cell proliferation and inhibits p53 protein expression. Our preliminary results provide a novel strategy to enhance cytotoxicity towards high-risk MDS cell line SKM-1.

## References

[1] Knipp S, Hildebrand B, Kündgen A, Giagounidis A, Kobbe G, Haas R, et al Intensive chemotherapy is not recommended for patients aged >60 years who have myelodysplastic syndromes or acute myeloid leukemia with high-risk karyotypes. Cancer. 2007; 110(2): 345–52. 17559141 10.1002/cncr.22779

[2] Kantarjian H, Beran M, Cortes J, O'Brien S, Giles F, Pierce S, et al Long-term follow-up results of the combination of topotecan and cytarabine and other intensive chemotherapy regimens in myelodysplastic syndrome. Cancer. 2006; 106(5): 1099–109. 16435387 10.1002/cncr.21699

[3] Xu B. Pharmacology of some natural products of China. Trends Pharmacol Sci. 1981; 2: 271–2.

[4] Xu B. The influence of several anticancer agents on cell proliferation, differentiation and the cell cycle of murine erythroleukemia cells. Am J Chin Med. 1981; 9(4): 268–76. 6964028 10.1142/s0192415x81000354

[5] Zhou JY, Chen DL, Shen ZS, Koeffler HP. Effect of homoharringtonine on proliferation and differentiation of human leukemic cells in vitro. Cancer Res. 1990; 50(7): 2031–5. 2317793

[6] Yao Y, Zhang G. Comparison of the effects of HA and DA regimens for adult acute non-lymphocytic leukemia. ACTA ACADEMIAE MEDICINAE XUZHOU. 1999; 19(3): 221–2.

[7] Jin J, Wang JX, Chen FF, Wu DP, Hu J, Zhou JF, et al Homoharringtonine-based induction regimens for patients with de-novo acute myeloid leukaemia: a multicentre, open-label, randomised, controlled phase 3 trial. Lancet Oncol. 2013; 14(7): 599–608. 10.1016/S1470-2045(13)70152-9 23664707

[8] Shu HE, Li WG, Ge SB. Analysis of HAG regimen in the treatment of 28 patients with MDS-RAEB. Zhongguo Wu Zhen Xue Za Zhi. 2007; 7(2): 331–2.

[9] Su JY, Chang CK, Zhang X, Zhou LY, Song LQ, Xu L, et al Efficacy of induction chemotherapy for patients with high-risk myelodysplastic syndrome (MDS) or MDS-transformed acute myeloid leukemia with CHG regimen and its comparison with regimen GAG and HA. Zhongguo Shi Yan Xue Ye Xue Za Zhi. 2009; 17(2):459–63. 19379588

[10] Wu L, Li X, Su J, Chang C, He Q, Zhang X, et al Effect of low-dose cytarabine, homoharringtonine and granulocyte colonystimulating factor priming regimen on patients with advanced myelodysplastic syndrome or acute myeloid leukemia transformed from myelodysplastic syndrome. Leuk Lymphoma. 2009; 50(9): 1461–7. 10.1080/10428190903096719 19672772

[11] Wu L, Li X, Su J, He Q, Zhang X, Chang C, et al Efficacy and safety of CHG regimen (low-dose cytarabine, homoharringtonine with G-CSF priming) as induction chemotherapy for elderly patients with high-risk MDS or AML transformed from MDS. J Cancer Res Clin Oncol. 2011; 137(10): 1563–9. 10.1007/s00432-011-1020-2 21845438 PMC11828265

[12] Braun T, Carvalho G, Coquelle A, Vozenin MC, Lepelley P, Hirsch F, et al NF-kappaB constitutes a potential therapeutic target in high-risk myelodysplastic syndrome. Blood. 2006; 107(3): 1156–65. 16223780 10.1182/blood-2005-05-1989

[13] Huang J, Ding T, Yang M, Liu H, Sun X, Jin J. Antitumor activity and drug interactions of proteasome inhibitor Bortezomib in human high-risk myelodysplastic syndrome cells. Int J Hematol. 2011; 93(4): 482–93. 10.1007/s12185-011-0821-z 21451957

[14] Eisfeld AK, Marcucci G, Maharry K, Schwind S, Radmacher MD, Nicolet D, et al miR-3151 interplays with its host gene BAALC and independently affects outcome of patients with cytogenetically normal acute myeloid leukemia. Blood. 2012(2); 120: 249–58. 10.1182/blood-2012-02-408492 22529287 PMC3398762

[15] Eisfeld AK, Schwind S, Patel R, Huang X, Santhanam R, Walker CJ, et al Intronic miR-3151 within BAALC drives leukemogenesis by deregulating the TP53 pathway. Sci Signal. 2014; 7(321): ra36 10.1126/scisignal.2004762 24736457 PMC4165404

[16] Riccioni R, Senese M, Diverio D, Riti V, Buffolino S, Mariani G, et al M4 and M5 acute myeloid leukaemias display a high sensitivity to Bortezomib-mediated apoptosis. Br J Haematol. 2007; 139(2): 194–205. 17897295 10.1111/j.1365-2141.2007.06757.x

[17] Li S, Pei R, Zhang P, Liu X, Ma J, Du X, et al Efficacy and safety of G-HAA regimen in treatment of refractory and relapsing acute myeloid leukemia. Zhejiang Medical Journal. 2013; 35(2): 105–10.

[18] Jin J, Jiang DZ, Mai WY, Meng HT, Qian WB, Tong HY, et al Homoharringtonine in combination with cytarabine and aclarubicin resulted in high complete remission rate after the first induction therapy in patients with de novo acute myeloid leukemia. Leukemia. 2006; 20(8): 1361–7. 16791270 10.1038/sj.leu.2404287

[19] Meng H, Yang C, Jin J, Zhou Y, Qian W. Homoharringtonine inhibits the AKT pathway and induces in vitro and in vivo cytotoxicity in human multiple myeloma cells. Leuk Lymphoma. 2008; 49(10): 1954–62. 10.1080/10428190802320368 18949618

[20] Chou TC, Talalay P. Quantitative analysis of dose-effect relationships: the combined effects of multiple drugs or enzyme inhibitors. AdvEnzyme Regul. 1984; 22: 27–55. 10.1016/0065-2571(84)90007-4 6382953

[21] Dlugosz PJ, Billen LP, Annis MG, Zhu W, Zhang Z, Lin J, et al Bcl-2 changes conformation to inhibit Bax oligomerization. EMBO J. 2006; 25(11): 2287–96. 16642033 10.1038/sj.emboj.7601126 PMC1478188

[22] Ryningen A, Ersvaer E, Øyan AM, Kalland KH, Vintermyr OK, Gjertsen BT, et al Stress-induced in vitro apoptosis of native human acute myelogenous leukemia (AML) cells shows a wide variation between patients and is associated with low BCL-2:Bax ratio and low levels of heat shock protein 70 and 90. Leuk Res. 2006; 30(12):1531–40. 16600371 10.1016/j.leukres.2006.02.014

[23] Cory S, Adams JM. The Bcl2 family: regulators of the cellular life-or-death switch. Nat Rev Cancer. 2002; 2(9): 647–56. 12209154 10.1038/nrc883

[24] Porter AG, Jänicke RU. Emerging roles of caspase-3 in apoptosis. Cell Death Differ. 1999; 6(2): 99–104. 10200555 10.1038/sj.cdd.4400476

[25] Crawford LJ, Irvine AE. Targeting the ubiquitin proteasome system in haematological malignancies. Blood Rev. 2013; 27(6): 297–304. 10.1016/j.blre.2013.10.002 24183816

[26] Nyåkern M, Tazzari PL, Finelli C, Bosi C, Follo MY, Grafone T, et al Frequent elevation of Akt kinase phosphorylation in blood marrow and peripheral blood mononuclear cells from high-risk myelodysplastic syndrome patients. Leukemia. 2006; 20(2): 230–8. 16341040 10.1038/sj.leu.2404057

[27] Karin M, Cao Y, Greten FR, Li ZW. NF-kappaB in cancer: from innocent bystander to major culprit. Nat Rev Cancer. 2002; 2(4): 301–10. 12001991 10.1038/nrc780

[28] Ben-Neriah Y, Karin M. Inflammation meets cancer, with NF-kappaB as the matchmaker. Nat Immunol. 2011; 12(8): 715–23. 10.1038/ni.2060 21772280

[29] Kloosterman WP, Plasterk RH. The diverse functions of microRNAs in animal development and disease. Dev Cell. 2006; 11(4): 441–50. 17011485 10.1016/j.devcel.2006.09.009

[30] Riley T, Sontag E, Chen P, Levine A. Transcriptional control of human p53-regulated genes. Nat Rev Mol Cell Biol. 2008; 9(5): 402–12. 10.1038/nrm2395 18431400

[31] Kruse JP, Gu W. Modes of p53 regulation. Cell. 2009; 137(4): 609–22. 10.1016/j.cell.2009.04.050 19450511 PMC3737742

[32] Hermeking H. p53 enters the microRNA world. Cancer Cell. 2007; 12(5): 414–8. 17996645 10.1016/j.ccr.2007.10.028

[33] He L, He X, Lowe SW, Hannon GJ. microRNAs join the p53 network—another piece in the tumour-suppression puzzle. Nat Rev Cancer. 2007; 7(11): 819–22. 17914404 10.1038/nrc2232 PMC4053212

[34] He L, He X, Lim LP, de Stanchina E, Xuan Z, Liang Y, et al A microRNA component of the p53 tumour suppressor network. Nature. 2007; 447(7148): 1130–4. 17554337 10.1038/nature05939 PMC4590999

